# The Three-Dimensional Investigation of the Radiographic Boundary of Mandibular Full-Arch Distalization in Different Facial Patterns

**DOI:** 10.3390/jpm14111071

**Published:** 2024-10-24

**Authors:** Yin-Yu Chou, Chia-Hsuan Chan, Yu-Jen Chang, Shiu-Shiung Lin, Chen-Feng Cheng, Te-Ju Wu

**Affiliations:** 1 Department of Orthodontics, Kaohsiung Chang Gung Memorial Hospital and Chang Gung, University College of Medicine, Kaohsiung 833253, Taiwan; joy126@cgmh.org.tw (Y.-Y.C.);; 2 Kaohsiung Municipal Feng-Shan Hospital, Kaohsiung 8330025, Taiwan; 3 Excellent Quality Dental Clinic, Kaohsiung 804608, Taiwan

**Keywords:** computed tomography, full-arch distalization, facial pattern, mandible

## Abstract

Objective: Mandibular full-arch distalization (MFD) is a popular approach, particularly in non-extraction cases. However, we still cannot confirm whether facial patterns affect the amount of limits. This study aimed to determine the anatomical MFD limits in patients with different facial patterns. Study design: Using computed tomography (CT), the shortest distances from the mandibular second molar to the inner cortex of the mandibular lingual surface and from the lower central incisor to the inner cortex of the lingual mandibular symphysis were measured in 60 samples (30 patients). The available distalization space in both regions was compared between groups with different facial patterns. Results: The available space in symphysis was more critical than that in retromolar area: the shortest distances to the inner cortex of the lingual mandibular symphysis at root levels 8 mm apical to the cementoenamel junction of the incisor were 1.28, 1.60, and 3.48 mm in the high-, normal-, and low-angle groups, respectively. Conclusions: Facial patterns affected the MFD capacity, and the thickness of the lingual mandibular symphysis was the most critical anatomic limit encountered. Practitioners should always pay attention to the possible impacts from facial patterns, especially in the treatment of high-angle cases.

## 1. Introduction

In recent years, with the development of temporary anchorage devices (TADs), full-arch distalization has become a popular approach for crowding relief and achieving appropriate overjet and overbite, particularly in cases expected to be treated by a non-extraction approach. Nevertheless, the anatomical limits always confine tooth movements.

The actual anatomical limit for full-arch distalization has long been of great interest in orthodontics. Recent studies have verified that the radiographic boundaries of molar distalization were the maxillary tuberosity in the maxilla [1,2,3], and the lingual cortex of the mandibular body under three-dimensional (3D) radiography [4,5,6,7,8]. Lee et al. [9] also reported that the average distalization capacity was 4.06 ± 1.93 mm in Class II maxilla and 2.80 ± 1.96 mm in Class III mandible. Distalization beyond the anatomical limit can lead to adverse effects such as root resorption, dehiscence, fenestration, and soft tissue recession [4,10,11]. Nerve numbness was also reported, compromising long-term oral health [12,13].

The goal of mandibular full-arch distalization (MFD) includes crowding relief and overjet correction in a non-extraction approach of Class III malocclusion. During the distalization movement, the anatomical limitation of both the lingual cortex of the mandibular body and lingual mandibular symphysis thickness must be considered. Moreover, vertical facial patterns are known to affect the available space over the two regions [4,14]. Horner et al. [15] reported that the thickness of cortical bone and alveolar ridge tended to be greater in hypodivergent subjects than in hyperdivergent subjects. For the anterior alveolus, the total thickness of the symphysis was greater in the low-angle subjects than in the high-angle subjects [16,17]. Furthermore, low-angle cases displayed thicker bone lingual to mandibular incisors [18,19].

However, to our knowledge, no studies have measured and compared the distalization limitation of the retromolar area with mandibular symphysis simultaneously. Thus, this study aimed to (1) determine the effect of different vertical facial patterns on the MFD boundary and (2) clarify the confounders correlated with MFD capacity, including sex, sides of the mandible, the existence of third molars, and vertical facial patterns.

## 2. Materials and Methods

The samples for this clinical study were recruited by retrospective screening of orthognathic cases between May 2015 and December 2022. This retrospective study was approved by the Institutional Review Board of Chang Gung Medical Foundation (IRB No. 202300179B0; Date of Approval: 2023/02/15). Presurgical medical multi-slice CT mandibular models were constructed, from which the posterior lingual boundary and lingual mandibular symphysis thickness were analyzed and measured with a computer-assisted system.

The inclusion criteria were as follows: age ≥ 18 years; healthy periodontal condition without noticeable alveolar bone loss; intact mandibular second molar roots without anomalies such as root resorption, hypercementosis, or severe dilacerations; absence of bony metabolism-related systemic diseases; without a history of orthodontic treatment; and without a history of craniofacial traumatic injury. The exclusion criteria were as follows: age < 18 years, missing mandibular second molar in any lower quadrant, presence of congenital craniofacial dysmorphology such as cleft lip or palate, history of mandibular resection or orthodontic treatment, earlier odontogenic tumor or pathosis, and presurgical CT of poor quality. Each patient’s mandibular model was divided into two segments (right and left), and each segment was regarded as an independent sample. Meanwhile, the wisdom tooth existence of each sample was also recorded.

To categorize the samples into different vertical facial patterns, the cephalometric module and some measurement parameters were utilized. One angular (S-N/Go-Me) and one linear (S-Go/N-Me) measured data were calculated to determine whether the samples belonged to the high-, normal- or low-angle group. For the S-Go/N-Me, <61% suggested a high angle, 61–69% indicated a normal angle and >69% signified a low angle [20]. For the S-N/Go-Me angle, <27° indicated a low angle, 27°–37° was a normal angle and >37° was a high angle [20]. If these two measurements did not point to the same category, or if the values were borderline, then the samples were abandoned.

### 2.1. 3D Image Reorientation

**Figure 1:**
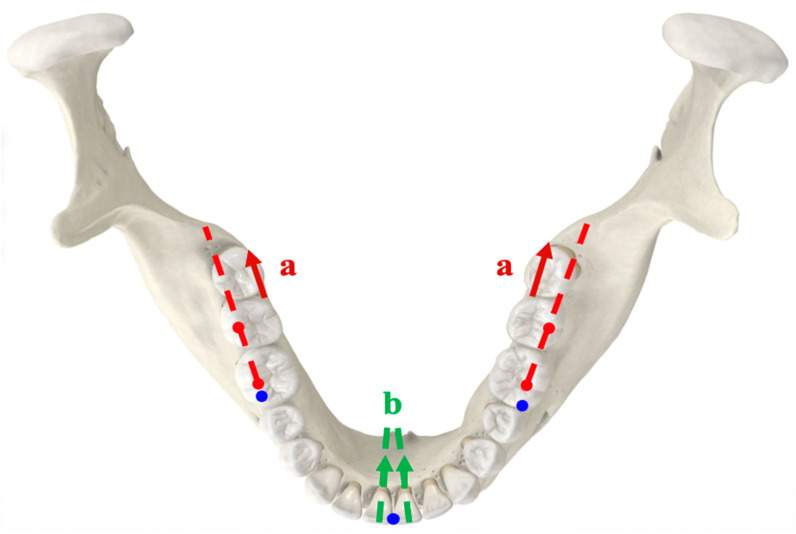
Reconstructed mandibular model with reference index. Blue dots: the three reference points constituting the mandibular occlusal plane. (**a**) Posterior lingual boundary: the shortest distance posterior to the mandibular second molar (dotted red line: the posterior occlusal line connecting the most occlusal point of the buccal grooves of the mandibular first and second molars). (**b**) Thickness of the lingual mandibular symphysis: the shortest distance lingual to the mandibular central incisor (dotted green line: the line perpendicular to the incisal edge from the occlusal view).

All patients were scheduled for a presurgical CT (Aquilion, Toshiba Corp., Tokyo, Japan) scan 1 month before orthognathic surgery, with the following parameters: 120 kVp; 350 mA; rotation time, 0.5 s; slice thickness, 0.5 mm. Rhinoceros 5.0 (Robert McNeel & Associates, Seattle, WA, USA) and Geomagic Studio (12th edition; Geomagic, Inc., Cary, NC, USA) were used for the 3D image processing and the orientation systems. The constructed 3D mandibular images were reoriented with the mandibular occlusal plane as a horizontal reference plane, connecting the midpoint of the mandibular right and left central incisor tips and the mesiobuccal cusps of the right and left mandibular first molars (Figure 1). The shortest linear distances from the mandibular second molar to the inner cortex of the mandibular lingual surface and from the lower central incisor to the inner cortex of the lingual mandibular symphysis were measured on axial slices parallel to this mandibular occlusal plane. The linear distances over the posterior area were measured parallel to the connecting line of the most occlusal point of the buccal grooves of the mandibular first and second molars (Figure 1). Conversely, the thickness of the lingual mandibular symphysis was measured in parallel with the line perpendicular to the incisal edge of the mandibular central incisor from the occlusal view (Figure 1).

### 2.2. CT Analysis for the Posterior Lingual Boundary

**Figure 2:**
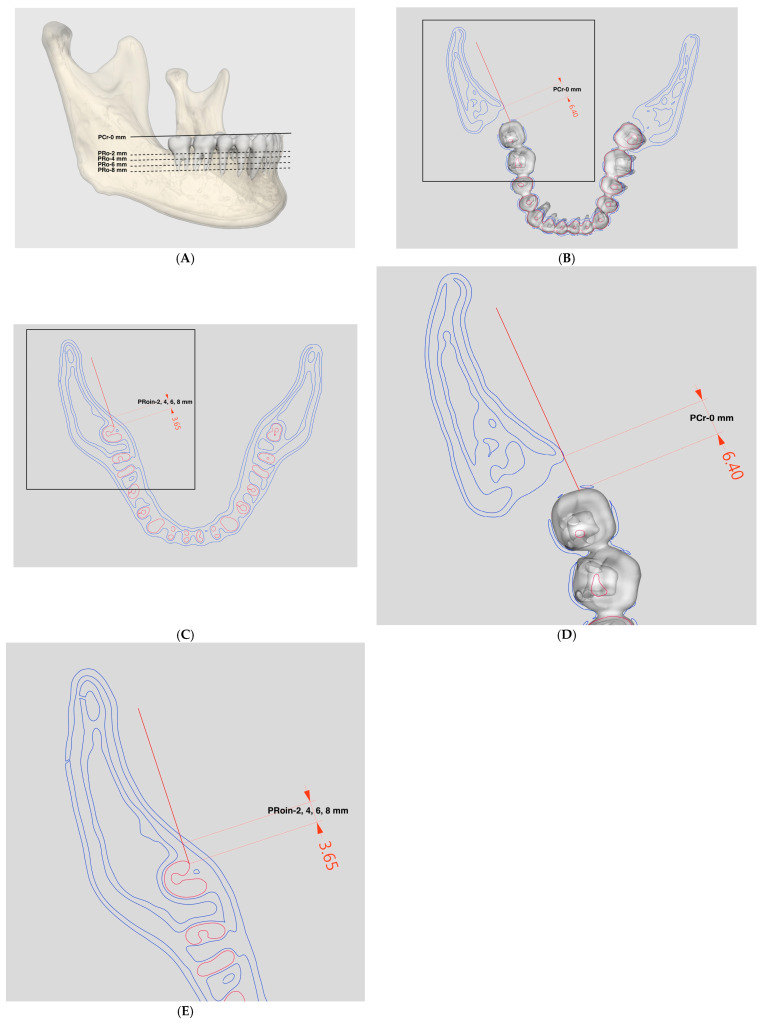
Posterior lingual boundary (in mm). (**A**). The axial slices at the depths of the crown-top level (solid gray line) and 2, 4, 6, and 8 mm apical to the CEJ (dotted gray lines) were made parallel to the designated mandibular occlusal plane. (**B**). The shortest distance from the mandibular second molar at the crown-top level to the outer cortex of the anterior surface of ramus (PCr-0 mm). (solid red line: the parallel index to the posterior occlusal line). (**C**). The shortest distance from the most lingual point of the distal root of the mandibular second molar to the inner cortex of the mandibular lingual surface at 2, 4, 6, and 8 mm apical to the CEJ (PRoin-2, -4, -6, and -8 mm, respectively). (solid red line: the parallel index to the posterior occlusal line) (**D**). Close-up view of the black box in B, showing the shortest distance to the outer cortex of the anterior surface of ramus (PCr-0 mm). (**E**). Close-up view of the black box in C, showing the shortest distance to the inner cortex of the mandibular lingual surface (PRoin-2, -4, -6, and -8 mm).

Measurements at the crown-top (Cr) and root (Ro) levels were performed on axial CT slices. The shortest distance from the mandibular second molar at the crown-top level to the outer cortex of the anterior surface of the ramus (PCr-0 mm) and from the most lingual point of the distal root of the mandibular second molar to the inner cortex of the mandibular lingual surface at 2, 4, 6, and 8 mm apical to the CEJ (PRoin-2, -4, -6, and -8 mm, respectively) were measured (Figure 2). The measured data were compared by sex, sides of the mandible, and vertical facial patterns, and their correlations were analyzed.

### 2.3. CT Analysis for the Lingual Mandibular Symphysis Thickness

**Figure 3:**
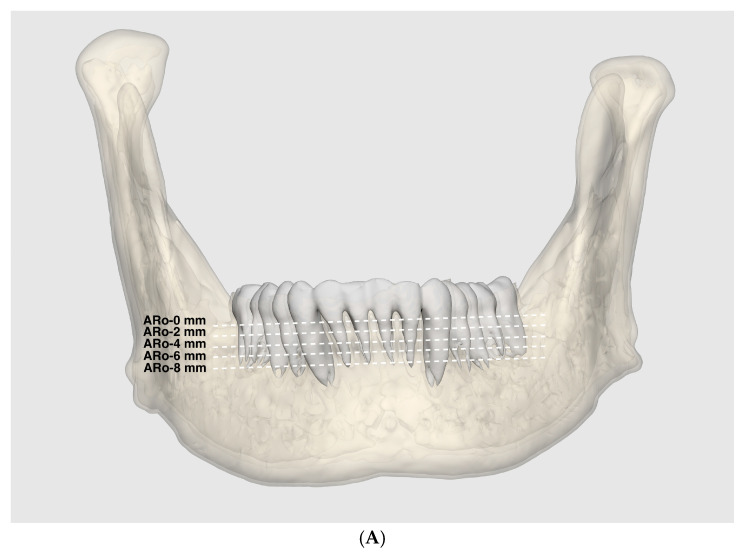

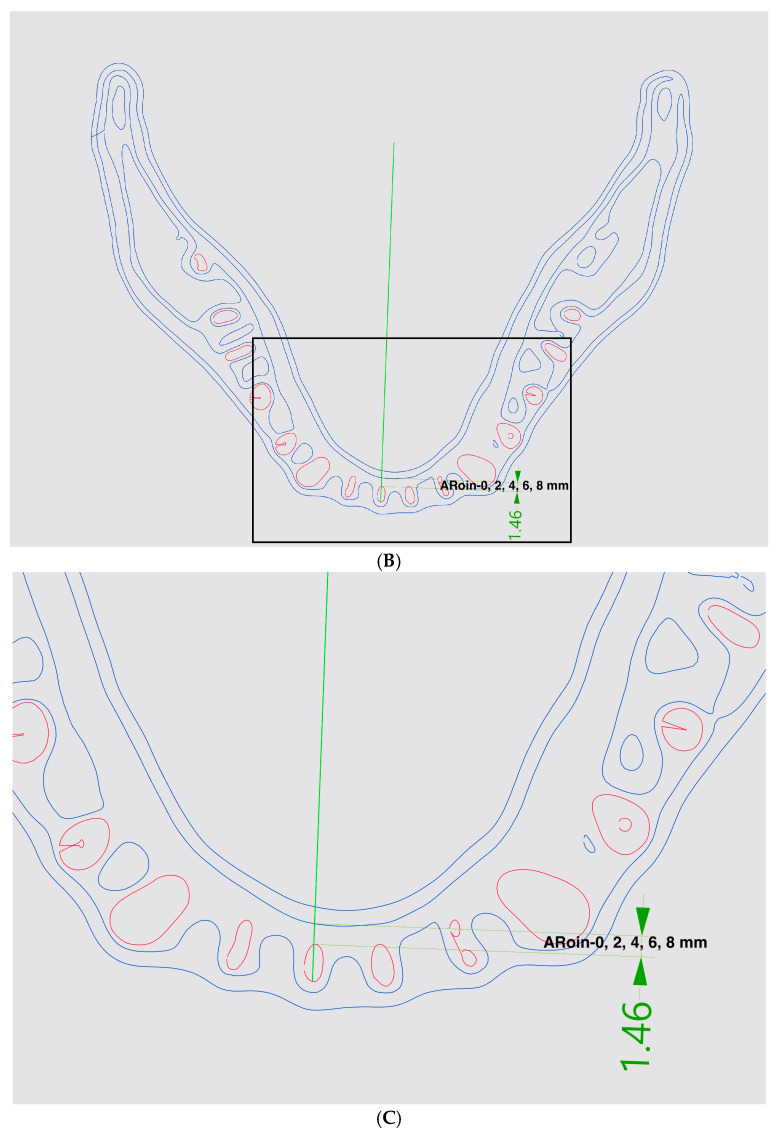
Thickness of the lingual mandibular symphysis (in mm). (**A**). The axial slices at depths of 0, 2, 4, 6, and 8 mm apical to the CEJ (dotted white lines) were made parallel to the designated mandibular occlusal plane. (**B**). Measurements of the shortest distance from the lingual point of the mandibular central incisor root to the inner cortex of the lingual mandibular symphysis at depths of 0, 2, 4, 6, and 8 mm apical to the CEJ on axial slices (ARoin-0, -2, -4, -6, and -8 mm, respectively). (solid green line: the parallel index perpendicular to the incisal edge of the mandibular central incisor). (**C**). Close-up view of the black box in B, showing the shortest distance to the inner cortex of the lingual mandibular symphysis (ARoin-0, -2, -4, -6, and -8 mm).

A similar approach was employed for analyzing the thickness of the lingual mandibular symphysis. The shortest distances from the lingual surface of the mandibular central incisor to the inner cortex of the lingual mandibular symphysis were measured at depths of 0, 2, 4, 6, and 8 mm apical to the CEJ on axial slices (ARoin-0, -2, -4, -6, and -8 mm, respectively) (Figure 3). As the posterior lingual boundary measurement, the thickness of the lingual mandibular symphysis was also compared and analyzed by sex, sides of the mandible, and vertical facial patterns.

### 2.4. Statistical Analysis

All landmarks on CT model imaging were manually located, with computer-assisted measurements applied simultaneously to calculate the distances. The obtained data were collected and analyzed using IBM SPSS Statistics for Windows version 25.0 (IBM Corp., Armonk, NY, USA). To assess intra-examiner consistency, all landmarks and distances were relocated and re-measured 2 weeks later by the same examiner and calculating system. The intra-class correlation coefficients (ICCs) and Dahlberg formula were used to evaluate measurement error. The ICC outcome was all > 0.95, and the Dahlberg error was all < 0.08 mm, suggesting high reproducibility of the landmark identification and digital measurement. The Shapiro–Wilk test was also used to evaluate data normality, and nonparametric statistics were performed in the subsequent analysis.

The independent-samples Mann–Whitney U-test was utilized to compare differences by sex (female vs. male) and sides of the mandible (right vs. left) in all samples. Additionally, the independent sample *t*-test was used to compare the distance to the radiographic boundary based on the existence or absence of third molars. Furthermore, the Kruskal– Wallis test was used to compare the differences in the posterior lingual boundary and lingual mandibular symphysis thickness in high-, normal-, and low-angle groups.

## 3. Results

Overall, 429 patients underwent orthognathic surgery at the Craniofacial Centre of Kaohsiung Chang Gung Memorial Hospital between May 2015 and December 2022. Among them, 226 patients had congenital craniofacial dysmorphology, 16 were traumatic cases, and 157 received presurgical orthodontic treatment, which were all excluded. The angular (S-N/Go-Me) and linear (S-Go/N-Me) parameters of the remaining samples all showed consistent classification in vertical facial patterns. Ultimately, 30 patients were enrolled in the experiment, for a total of 60 datasets. Patients’ characteristics are shown in Table 1. The samples consisted of 12 males and 18 females (age range, 18–36; mean age, 23.5 ± 4.1 years). From the measurement, the distalization capacity in both the posterior lingual area and mandibular symphysis were not significantly different regardless of sex and sides of the mandible (Table 2). Thus, data from both sexes and sides were independently distributed to three vertical facial pattern groups for subsequent analysis. The mean age and sex distributions were not significantly different in the three groups (*p* > 0.05) (Table 1).

### 3.1. Difference in the Posterior Lingual Boundaries in the High-, Normal-, and Low-Angle Groups

The shortest distance from the mandibular second molar at the crown-top level to the outer cortex of the anterior surface of the ramus (PCr-0 mm) and from the distal root of the mandibular second molar to the inner cortex of the mandibular lingual surface at 2, 4, 6, and 8 mm apical to the CEJ (PRoin-2, -4, -6, and -8 mm, respectively) are shown in Table 3. All mandibular second molar roots were no longer observed at 10 mm apical to the CEJ level. The results indicated that the available distalization space of the mandibular second molar was getting restricted as it got close to the apical area. That is, the anatomical limit posterior to the mandibular second molar was probably located nearby the inner cortex at root level 8 mm apical to the CEJ. The distances were 2.45, 3.64, and 4.39 mm in high-, normal-, and low-angle groups. And the Kruskal–Wallis test indicated that the distances among three facial patterns were significantly different at all axial levels (*p* < 0.05) (Table 3), with less space availability in the high-angle group and greater space in the low-angle group in the posterior lingual boundary. However, the existence of the third molar seems unrelated to the amount of available retromolar space (Table 4).

### 3.2. Difference in the Thickness of Lingual Mandibular Symphysis in High-, Normal-, and Low-Angle Groups

The shortest distance from the lingual surface of the mandibular central incisor to the inner cortex of the lingual mandibular symphysis at depths of 0, 2, 4, 6, and 8 mm (ARoin-0, -2, -4, -6, and -8 mm, respectively) apical to the CEJ in three facial patterns are presented in Table 3, and all central incisor roots were no longer seen at 10 mm apical to the CEJ. Contrary to the posterior lingual boundary, the thickness of the lingual mandibular symphysis tended to increase as it approached the apex. The shortest distance to the inner cortex of the lingual mandibular symphysis at root levels 8 mm apical to the CEJ were 1.28, 1.60, and 3.48 mm in high-, normal-, and low-angle groups, respectively. Statistical comparison in the thickness of lingual mandibular symphysis showed significant difference among the three groups at the inner limits of root levels 4, 6, and 8 mm apical to the CEJ (ARoin-4, -6, and -8 mm). Low-angle cases presented thicker lingual mandibular symphysis than normal and high-angle cases except for root levels 0 and 2 mm apical to the CEJ (ARoin-0 and -2 mm). Although there was a minimal difference, the existence of the third molar was related to the increased distance to the anterior radiographic boundary in the coronal one-third of the high-angle samples and in the cervical region of the ones with a low facial angle (Table 4).

## 4. Discussion

In this study, we found it was the inner surface of the lingual mandibular symphysis, rather than the lingual cortex of the mandibular body, the true anatomical limit encountered during MFD in cases not requiring crowding relief. Moreover, the distalization capacity between sex and sides of the mandible in the posterior lingual area and mandibular symphysis were not significantly different (Table 2). However, vertical facial patterns did affect the distalization capacity: people with a high-angle tended to have thinner lingual mandibular symphysis and restricted posterior lingual area, whereas those with a low-angle were usually born with a broader distalization space. Therefore, for crowding relief in the non-extraction approach, people with a high angle tended to encounter a space shortage in the molar area during MFD. On the contrary, for non-crowded cases, orthodontists should be aware of the thickness of the lingual mandibular symphysis during MFD regardless of vertical facial patterns.

The MFD capacity over the posterior lingual area and lingual mandibular symphysis tended to decrease as the vertical facial angle increased. The shortest distances from the distal root of the mandibular second molar to the inner cortex of the mandibular lingual surface were 2.45, 3.64, and 4.39 mm in the high-, normal-, and low-angle groups. Conversely, the shortest distances at the same level from the lingual surface of the mandibular central incisor to the inner cortex of the lingual mandibular symphysis were 1.28, 1.60, and 3.48 mm in the high-, normal-, and low-angle groups, respectively. The median discrepancy can be up to 1.94 mm in posterior lingual area and 2.2 mm in lingual mandibular symphysis between the high- and low-angle groups near the apex. This discrepancy in the posterior lingual boundary in our study was similar to that in another Korean study, being 2.08 mm between the high- and low-angle groups [4]. However, in the thickness of lingual mandibular symphysis at the apex, the discrepancy between the high- and low-angle groups was only 0.3–0.6 mm in other studies under cone-beam CT (CBCT) and lateral cephalometric films [17,21]. The various results may be due to the different image resolutions of examination instruments and the different races or malocclusion types of samples recruited.

Several studies had tried to determine the association of vertical facial pattern and MFD capacity and presented some explanation. First, with the same horizontal mandibular body length, a high-angle pattern would contribute to a geometrically shorter distance of the posterior mandibular dimension and a thinner lingual mandibular symphysis [4]. Second, individuals with a high-angle pattern usually showed more dentoalveolar compensation by the eruptive movement of the mandibular molars and incisors to coordinate with its vertical growth tendency [21,22]. The alveolar bone remodeled with the dental extrusion, becoming higher and thinner over both the posterior lingual area and mandibular symphysis [21,22]. Third, patients with a low-angle pattern tended to have a stronger masticatory force than those with a high-angle pattern, resulting in more accumulative periosteal bone deposition and thicker bone volume over the mandibular lingual surface than those with high and normal-angle patterns [23,24]. Some researchers also reported that genetic factors were related to the vertical facial patterns contributing to various distalization extremities [4,25].

It is worthy of notice, although the sample power in the low-angle subgroup was relatively limited. Our findings, which showed less MFD capacity in the high-angle group and more capacity in the low-angle group, were aligned with previous reports [4,14,26]. Furthermore, we also found that when comparing the anterior and posterior limits simultaneously, the actual MFD limitation would be the inner surface of the lingual mandibular symphysis rather than the lingual cortex of the mandibular body. In those high-angle cases, the limits could be minimized to a mean value of 1.28 mm. Thus, high-angle individuals may take a higher risk of negative consequences on teeth against surrounding alveolar bone during MFD, which restricted the implementation of Class III camouflage treatment. This result corresponded to the surgical property of our samples.

Several studies agreed with such a perspective. Wehrbein H et al. [27] demonstrated that a mandible with a narrow and high symphysis, which was common in high-angle cases, went through progressive loss of both the buccal and lingual bone when the lower incisors were moved to the lingual direction and de-rotated because of thinner alveolar bone support. Under CBCT, Hoang et al. [28] also reported that the high-angle group was at risk of external root resorption of the mandibular incisors, with an incidence of 22% compared with 20% in the average-angle group and 9% in the low-angle group. Moreover, the high-angle group had a higher risk for incisors moving out of the bone housing, either buccally or lingually, during orthodontic treatment, with an incidence of 22% compared with 5% and 0% in the average- and low-angle groups, respectively [28].

To prevent those sequelae, the alveolar bone housing should always be considered during MFD [29,30]. Clinically, excessive molar lingual root torque should be prevented to keep the roots away from the mandibular lingual cortex. For example, we should avoid the overbent reverse curve of Spee in those cases treated by high-torque-prescription brackets. Meanwhile, Sung-Ho Kim et al. [4] also suggested stiffer arch wires with some lingual crown torque during buccal TAD-facilitated MFD to prevent uncontrolled buccal crown-tipping of the posterior teeth. Likewise, molar intrusive movements would bring the root apices closer to the lingual cortex and thus should be avoided. Regarding the mandibular incisors, some lingual crown-tipping and intrusion are preferred to create longer distalization lengths between the incisor apices and inner cortex of the lingual mandibular symphysis. Conclusively, appropriate mechanics should be planned before performing MFD.

**Figure 4:**
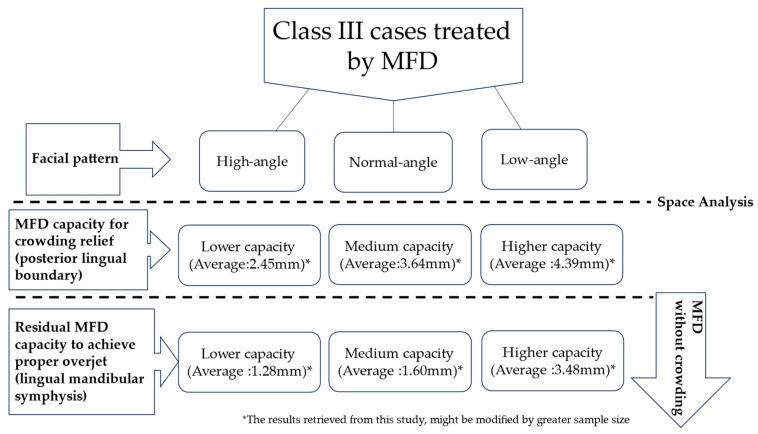
A simplified diagram summarizing the thinking process to evaluate the treatment strategies in patients of Class III malocclusion among different facial patterns.

Also, treatment strategies for crowding relief may vary according to vertical facial patterns. As low-angle individuals are usually born with spacious bone housing, treatment strategies to regain by distalization or arch expansion could be more feasible. On the other hand, more invasive approaches such as extraction or interproximal reduction shall be considered for high-angle cases. It’s always important for practitioners to realize the bony boundary before making any dental movement. Orthodontists should adapt their techniques based on different facial patterns (Figure 4).

Additionally, the existence of mandibular third molars might be another issue relating to the MFD capacity. Previous studies showed conflicting results on whether the existence of third molars yielded greater alveolar volume and provided a favorable environment for mandibular molar distalization [4,5,31]. However, in our study, the existence of third molars was not associated with increased distance between the second molar and posterior boundary (Table 4). It would be because of the relatively minor changes in the mandibular lingual cortex compared to the noticeable resorption on the buccal side after the extraction of the mandibular third molar [32,33,34,35].

Besides the bone structure limitations, soft tissue impingement should be considered during MFD. The soft tissue overlying the retromolar area sometimes hinders tooth movement or compromises post-distalization stability [4]. Inflammation can frequently occur if the dental crown is partially covered by the soft tissue or if insufficient gingiva is attached [4,36]. To overcome those difficulties, some patients will need gingivectomy and even slight bone contouring over retromolar areas. For the mandibular incisor area, the soft tissue is usually thin and rarely needs reduction surgery; however, interdental dark triangles may appear after distalization if the original tooth morphology or periodontal condition are unfavorable [37,38].

Another concern is the inferior alveolar nerves and vessels; the superior border of the inferior alveolar canal is usually adjacent to and may restrict the distalization of the mandibular second molar root at the apex [6,8,39]. More seriously, temporary lip paraesthesia during mandibular molar distalization was reported [12,13]. Therefore, a careful 3D morphological survey is very important to distinguish those structures and obtain a comprehensive understanding before such orthodontic movements.

### 4.1. Limitations

This study had some shortcomings. First, although the power of the overall sample size has exceeded the minimal requirement, the sample size for each subgroup remained limited, especially in the low-angle subgroup. We expect to extend our survey to the non-surgical samples to have more solid evidence. Second, the inclinations of the mandibular second molars and central incisors were not measured, which would also influence the distance from the root apex to the inner cortex. Third, alveolar bone remodeling and morphological change during orthodontic movement remained unpredictable and could be hardly simulated. More detailed investigations of the related confounding factors and 3D spatial analysis were warranted.

### 4.2. Expectations

Based on the CT-constructed models and computer-assisted measurement system, currently, we can distinguish meticulous structures and avoid errors from manual measurement, thus enhancing the reproducibility and reliability of the resulting data. In the future, landmark labeling can serve as a critical input in artificial intelligence (AI) studies. The results and significant measurements might become valuable features in subsequent studies that apply AI models to provide timely information in a clinical environment.

## 5. Conclusions

The following conclusions were derived from this research:

1. Vertical facial patterns indeed affect the distalization capacity; people with a high-angle pattern are susceptible to reduced MFD capacity, either in the posterior lingual area or lingual mandibular symphysis. While sex or sides of the mandible would not affect the MFD capacity.
2. Regardless of facial patterns, lingual mandibular symphysis is the true anatomical limit encountered during MFD in non-crowded cases.
3. Orthodontists should be cautious about space management and MFD capacity based on specific facial patterns before treatment and use appropriate biomechanics during treatment to prevent teeth from violating anatomical limits.

## Figures and Tables

**Table 1:** Patient characteristics.

	High-Angle Group	Normal-Angle Group	Low-Angle Group	*p* Value
No. of patients, n	10	14	6	0.623 ^a^
No. of samples, s	20	28	12	
Sex distribution (male/female)	3 (30%)/7 (70%)	7 (50%)/7 (50%)	2 (33.3%)/4 (66.7%)	
Age (50th percentile)	24.4	23.3	22.6	0.519 ^b^
Age (standard deviation)	3.6	4.9	2.2	

^a^. Statistical significance for the difference in sex distribution among the high-, normal-, and low-angle groups, tested using Fisher’s exact test (statistically significant, *p* < 0.05). ^b^. Statistical significance for the difference in age distribution among the high-, normal-, and low-angle groups, tested using the Kruskal–Wallis test (statistically significant, *p* < 0.05).

**Table 2:** Comparison between groups by sex and sides of the mandible (in mm).

Depth	*p*-Value Between Male and Female (Statistically Insignificant, *p*-Value All > 0.05)	*p*-Value Between the Right and Left Sides of the Mandible (Statistically Insignificant, *p*-Value All > 0.05)
PCr-0 mm	0.519	0.575
PRoin-2 mm	0.214	0.739
PRoin-4 mm	0.840	0.722
PRoin-6 mm	0.244	0.306
PRoin-8 mm	0.155	0.324
ARoin-0 mm	0.952	0.383
ARoin-2 mm	0.437	0.610
ARoin-4 mm	0.931	0.726
ARoin-6 mm	0.297	0.721
ARoin-8 mm	0.475	0.784

Abbreviations: PCr-0 mm, The shortest distance between the mandibular second molar crown-top and the outer cortex of the anterior ramus. PRoin-2, -4, -6, -8 mm, The shortest distance from the most lingual point of the distal root of the mandibular second molar to the inner cortex of mandibular lingual surface at 2, 4, 6, 8 mm apical to the CEJ. ARoin-0, -2, -4, -6, -8 mm, the shortest distance from the lingual surface of mandibular central incisor to the inner cortex of lingual mandibular symphysis at depths of 0, 2, 4, 6, 8 mm apical to the CEJ.

**Table 3:** Descriptive analysis and statistical comparison of the posterior lingual boundary and thickness of the lingual mandibular symphysis between the crown and different root levels in high-, normal-, and low-angle adult groups (in mm).

Depth	Vertical Facial Pattern	Minimum	Median 50%	Maximum	*p*-Value
Crown top level	Shortest distance to the outer cortex of the anterior ramus				
PCr-0 mm	High-angle	6.70	9.6525	14.85	0.004 *
	Normal-angle	7.02	11.3100	14.23	
	Low-angle	10.02	12.9925	14.41	
Root level apical to CEJ	Shortest distance to the inner cortex of the mandibular lingual surface				
PRoin-2 mm	High-angle	1.78	5.1000	7.65	0.043 *
	Normal-angle	3.46	6.0200	7.69	
	Low-angle	3.53	6.2100	8.63	
PRoin-4 mm	High-angle	1.36	4.5550	6.23	0.003 *
	Normal-angle	3.00	5.4400	7.37	
	Low-angle	3.75	6.1800	7.78	
PRoin-6 mm	High-angle	1.57	3.6100	5.44	0.001 *
	Normal-angle	2.32	4.2250	5.34	
	Low-angle	2.73	5.3625	6.64	
PRoin-8 mm	High-angle	2.15	2.4450	3.56	0.035 *
	Normal-angle	1.69	3.6400	5.18	
	Low-angle	2.52	4.3850	5.59	
Root level apical to CEJ	Shortest distance to the inner cortex of the lingual mandibular symphysis				
ARoin-0 mm	High-angle	0.32	0.4525	0.52	0.334
	Normal-angle	0.10	0.4125	0.58	
	Low-angle	0.08	0.3750	0.61	
ARoin-2 mm	High-angle	0.35	0.4875	0.84	0.983
	Normal-angle	0.11	0.5000	0.94	
	Low-angle	0.12	0.5200	1.25	
ARoin-4 mm	High-angle	0.33	0.4875	1.16	0.004 *
	Normal-angle	0.31	0.5675	1.24	
	Low-angle	0.52	0.8025	2.03	
ARoin-6 mm	High-angle	0.35	0.7000	1.99	0.001 *
	Normal-angle	0.47	1.0625	3.68	
	Low-angle	0.69	2.0175	3.63	
ARoin-8 mm	High-angle	0.71	1.2750	1.67	0.001 *
	Normal-angle	0.56	1.5950	4.84	
	Low-angle	2.03	3.4800	5.21	

Intergroup comparison; *: Statistically significant, *p* < 0.05. Abbreviations: PCr-0 mm, The shortest distance between the mandibular second molar crown-top and the outer cortex of the anterior ramus. PRoin-2, -4, -6, and -8 mm, The shortest distance from the most lingual point of the distal root of the mandibular second molar to the inner cortex of mandibular lingual surface at 2, 4, 6, and 8 mm apical to the CEJ. ARoin-0, -2, -4, -6, and -8 mm, the shortest distance from the lingual surface of mandibular central incisor to the inner cortex of lingual mandibular symphysis at depths of 0, 2, 4, 6, and 8 mm apical to the CEJ. CEJ, cementoenamel junction.

**Table 4:** Descriptive analysis and statistical comparison of the anterior and posterior radiographic boundary in high-, normal-, and low-angle adult groups (in mm) based on the presence or absence of the third molars.

Group		High Angle Facial Pattern				Normal Angle Facial Pattern				Low Angle Facial Pattern			
Items	3rd molar ^#^	N	AVG ^$^	SEM ^%^	*p* ^+^	N	AVG ^$^	SEM ^%^	*p* ^+^	N	AVG ^$^	SEM ^%^	*p* ^+^
ARoin_0 mm	0	10	0.41	0.02	0.350	15	0.36	0.04	0.143	9	0.27	0.05	0.029 *
	1	10	0.44	0.02		13	0.44	0.03		3	0.53	0.07	
ARoin_2 mm	0	10	0.45	0.02	0.033 *	15	0.47	0.07	0.443	9	0.44	0.12	0.151
	1	10	0.57	0.05		13	0.53	0.03		3	0.79	0.14	
ARoin_4 mm	0	10	0.45	0.03	0.008 *	15	0.66	0.08	0.626	9	0.92	0.15	0.551
	1	10	0.71	0.08		11	0.62	0.06		3	1.11	0.31	
ARoin_6 mm	0	10	0.65	0.07	0.053	15	1.19	0.15	0.657	9	2.00	0.35	0.605
	1	10	1.04	0.17		11	1.34	0.33		3	2.38	0.63	
ARoin_8 mm	0	4	0.95	0.15	0.145	10	2.07	0.39	0.891	9	3.42	0.34	---
	1	5	1.31	0.16		8	1.98	0.55		1	3.66	---	
PRoin_2 mm	0	10	5.07	0.52	0.967	10	5.07	0.52	0.967	9	6.14	0.41	0.296
	1	10	5.04	0.34		10	5.04	0.34		3	7.19	1.16	
PRoin_4 mm	0	10	4.22	0.42	0.643	10	4.22	0.42	0.643	9	5.64	0.41	0.330
	1	10	4.46	0.28		10	4.46	0.28		3	6.55	0.95	
PRoin_6 mm	0	10	3.61	0.34	0.498	10	3.61	0.34	0.498	9	4.72	0.40	0.248
	1	10	3.35	0.17		10	3.35	0.17		3	5.73	0.758	
PRoin_8 mm	0	2	2.23	0.08	0.272	2	2.23	0.08	0.272	9	4.16	0.89	---
	1	7	2.65	0.18		7	2.65	0.18		0	--	---	

^#^ 0: none, 1: existed; ^$^: Average; ^%^: Standard error of mean; ^+^: *p* value; *: *p* value < 0.05.

## Data Availability

The raw data supporting the conclusions of this article will be made available by the authors upon request.
